# Food exchange list based on macronutrients: adapted for the Ecuadorian population

**DOI:** 10.3389/fnut.2023.1219947

**Published:** 2023-08-10

**Authors:** Aida Maribel Chisaguano-Tonato, María Elisa Herrera-Fontana, Gabriela Vayas-Rodriguez

**Affiliations:** ^1^Universidad San Francisco de Quito USFQ, Colegio de Ciencias de la Salud, Nutrición y Dietética, Quito, Ecuador; ^2^Universidad San Francisco de Quito USFQ, Colegio de Comunicación y Artes Contemporáneas, Quito, Ecuador

**Keywords:** food exchanges, exchange portions, dietary planning, Ecuador, serving sizes, macronutrients

## Abstract

**Background:**

Food exchange lists allow health professionals to generate healthy eating plans adapted to individual or population needs. The objective of this study was to develop the first food exchange list based on the macronutrients and energy provided by the various food groups of the Ecuadorian diet.

**Methods:**

The list of Ecuadorian food exchanges was constructed by going through the following phases: (1) Selection of household measurements; (2) Selection of tables and databases of the nutritional composition of food items; (3) Definition of food groups and quantities; (4) Determination of the average energy and macronutrient values of each group; and (5) Photographic record. For the definition of food quantities, statistical criteria were applied according to a standard deviation of ±2SD; thus, for carbohydrates: ±5 g, total fat: ±2 g, and protein: ±3 g. To ensure the inclusion of the food items in the groups, a coefficient of variation of less than 30% and a Z value of ±2 were also considered.

**Results:**

The list of food exchanges is presented with eight general groups according to the predominant nutrient (carbohydrates, proteins, or fats), and, where necessary, subgroups are included according to the second predominant nutrient. The list includes 404 food items with their photographic record, represented by their net weights and corresponding household measurement. All food items met the statistical criteria that help to reduce the variability of the nutritional composition of the food items in each group.

**Conclusion:**

This is the first list of Ecuadorian food exchanges based on statistical criteria. It represents a novel tool for public health professionals as well as researchers. Resulting healthier eating plans may improve daily dietetic practice, facilitate better clinical trial designs and help establish guidelines according to Ecuador’s cultural and dietary patterns. The described methodology can further be used to develop other food exchanges lists for patients with specific nutritional requirements.

## 1. Introduction

Food exchange lists or systems are educational tools that have allowed nutrition and dietetic professionals to plan healthy meals without altering the macronutrient or energy content in a fast, practical, and reliable manner according to people’s tastes and habits and that meet nutritional requirements (1). The exchange lists were originally created for the nutritional management of patients with diabetes, who are required to control their carbohydrate intake (CHO) since the CHO count is used to plan their meals and focuses on estimating the macronutrient content that mainly influences the postprandial glycemic response (2). However, nowadays the use of the lists has been extended to other metabolic pathologies, such as obesity or cardiovascular diseases (3). In addition, lists have been developed for populations with specific needs, such as athletes (4), vegans (5) or those using supplementation for malnutrition (6).

Furthermore, food exchange lists allow experts to consider the reality of each country in order to develop appropriate food guides and nutritional education programs at local and national levels for the prevention and control of diseases. The lists guarantee individuals’ and target communities’ welfare and nutritional requirements, as applied in countries such as the United States (7), Spain (8), Greece (9), Jordan (10), Lebanon (11), and Sri Lanka (12). In Latin America, few countries have achieved the construction of exchange lists adapted to their cultural and dietary patterns, thus neighboring countries such as Peru (13), Colombia (14), or Chile (15) have acted as references in the region. In Ecuador, these exchange lists have been used both in clinical practice and in the training of nutrition professionals. However, a national food exchange list in Ecuador has not been available previously due to a lack of reliable information on the nutrient composition of many food items, as well as the scarcity of data concerning serving sizes as standard kitchen measurements (spoons, cups, glasses, etc.) and their weight in grams. Nevertheless, there is already a record of the most commonly used household measurements in the country (16).

The absence of a national exchange list has made it difficult for nutritionists working with Ecuadorian patients suffering from obesity or other chronic diseases to diversify their diets. In fact, Ecuadorian nutritionists still use food exchange lists from Mexico (17) and the United States (18), even though the use of an external list increases the risk of introducing possible biases, especially those related to dietary patterns since these are specific to each country. Thus, the lack of a national food exchange food has limited the design and creation of dietary plans at various levels of care, the development of research projects, and the generation of dietary guidelines or programs.

Therefore, the objective of this study was to build the first food exchange list in Ecuador, ensuring that it respects the country’s food culture, according to macronutrient and energy contents. It also enables the conversion of each food item’s weight measurements with the most commonly used household measurements in the country, supported by the photographic record that facilitates the use of the tool.

## 2. Materials and methods

The number of food items, portion sizes, and photographic record were defined according to the following phases.

### 2.1. Phase 1: selection of household measurements

Given that one of the objectives of the exchange lists is the management of quantities in grams through the use of household measurements, for this study, those measurements were selected by considering two stages, as follows. (i) *The most frequently sold household measurements in key retail centers, such as hypermarkets and specialized stores, were recorded and a preliminary list was set out*. At this stage, a list of the household measurements most frequently sold by the retail center was requested. Based on that, the 10 most frequently sold measurements in each category were chosen and a preliminary photographic record was made. (ii) *The preliminary list was evaluated by a group of experts in food intake recording*. At this stage, nutritionists and experts in dietary intake from different regions of the country (the coastal, Highland, and Amazon regions) were contacted and they voluntarily agreed to evaluate the preliminary list with the respective images. On this matter, the experts were asked to choose the measurement or measurements most used in their population through the images provided.

Finally, the experts’ answers were contrasted, the criteria were unified with the research group, and the most commonly used household measurements in the country were defined.

### 2.2. Phase 2: selection of the tables and databases of the nutritional composition of food items

The list of food items and nutrients was selected according to the food and culinary culture and eating habits of the Ecuadorian diet, based on the food consumption database from the National Health and Nutrition Survey (ENSANUT 2011–2013) (19). Food items selected from the general database were included according to region and sex.

The nutritional information of food items was obtained using the food composition tables (FCT) from countries in the region. Data were obtained mainly from eight information sources, where the Central American food composition table (20) was the main reference source. For those food items not found in this table, other sources were used: FoodData Central in the United States (21); Peruvian food composition tables (22); Colombian food composition table, 1st and 2nd ed. (23, 24); food composition table in Cuenca, Ecuador (25); standardized recipes, nutritional labeling, and scientific articles (26).

Once the nutritional composition of each food was established, an analysis of variability was performed to detect the variation between individuals according to the following nutrients: energy, carbohydrates, proteins, fats, iron, calcium, zinc, and vitamins A and C. Therefore, the preliminary list included food items that are frequently consumed and those that provide critical nutrients for the Ecuadorian population.

### 2.3. Phase 3: definition of food groups and quantities

The exchange list was constructed by considering the food groups recommended by FAO and WHO in the Codex Alimentarius (27, 28). In order that the amount of each food item could be interchanged with any other food within the same group without presenting significant differences in its nutritional value, different amounts of each item were entered into an Excel database that enabled us to compare the value of energy and macronutrients until we reached the most appropriate amount, according to the established statistical parameters.

In all cases, for each food item, the amount in grams was evaluated according to Ecuadorian dietary and culinary practices and, when necessary, we followed the recommendations of the national guidelines INEN 1334-2 that regulates the portion size for packaged food products (29). Subsequently, the quantity was also estimated using the usual household measurements of the Ecuadorian population (16).

For each food group, food items whose amounts met the statistical criteria for macronutrients were included. The values established by Wheeler et al. (30) were used, taking as a reference the representative macronutrient for each food group, where a standard deviation of ±2SD was considered, i.e., ±5 g for carbohydrates, ±2 g for total fat, and ± 3 g for protein, as appropriate. Furthermore, once the criteria for macronutrients were met, the energy and caloric value of each food item was calculated by multiplying the carbohydrate (4 kcal/g), fat (9 kcal/g), and protein (4 kcal/g) content by their respective Atwater factors (8). If the SD value was outside these limits, the food item was removed from the group and relocated to a more appropriate group. Once the SD was adjusted, the coefficient of variation (CV) was estimated to be less than 30%. In addition, for those groups in which the CV was high, the Z value for each food was calculated in order to eliminate foods with high variation. The *Z* value was considered to be ±2. This statistical criterion was applied to homogenize the number of food items within each group (8).

Once the quantities were defined in the database, raw and cooked weights were recorded for all food items in an experimental kitchen, using an electronic scale (METTLER TOLEDO, 0.5–3,100 g; ±0.01 g), depending on the most common form of consumption. A triplicate record was applied to each measurement [(weight (g), length (cm), width (cm), and height (cm)] to reduce measurement variability.

### 2.4. Phase 4: determination of the average macronutrient and energy values for each food group

The macronutrient values for each exchange group correspond to the average in grams of the food list in each group and are presented as whole numbers (values less than 0.5 were rounded down and values greater than 0.5 were rounded up). Finally, in the case of the energy of each exchange group, the values correspond to the average amount in kilocalories (kcal) and were rounded to the nearest ten; for example, for the low-carbohydrate fruit group, an average intake of 47 kcal was recorded, which was rounded up to 50 kcal in order to present easy-to-remember numbers for the practitioner.

### 2.5. Phase 5: photographic recording

A professional photographer took photographs of all the food items, It is noted that a professional photographic set was organized next to the food preparation area to keep the food fresh, thus simulating a real-life consumption situation.

For this, we worked with a professional camera (Canon EOS 7D) and a lighting scheme for products, which consisted of two softbox lights with flash triggers (Hensel Integra 500), a zenithal side light as the main light and a semi-zenithal side light as a secondary light, plus a silver reflective bouncer to help reduce shadows and maintain the volume. An ISO of 100 was used, so as not to produce marked grain detail, along with a 70 mm medium telephoto lens (Zeiss Canon Mount) so as not to produce any distortion, and an average camera angulation of 50 degrees. In addition, a grayscale color pattern was used for the background to facilitate the focus on the food rather than the surroundings.

Due to the great variety of food ítems and their shapes, colors, and arrangement on the plate or household measurement used, the focal distance between the camera and the food varied between 99 and 138 cm, and for the height of the tripod a range of 146–163 cm was used.

In this research, the aim of the photography was not to beautify the food, as happens in the commercial field. On the contrary, what was sought was for the food to look natural and be perceived as if the consumer was seeing it in order to consume it. This concept was the driving force for the inclination of the camera and the technical decisions of the photography (31). It was necessary to change the framing and settings on several occasions due to the type of food and household measurement that contained it, because it is different to, for instance, photograph a food item in a cup than one in a spoon.

## 3. Results

### 3.1. Household measurements

**Figure 1:**
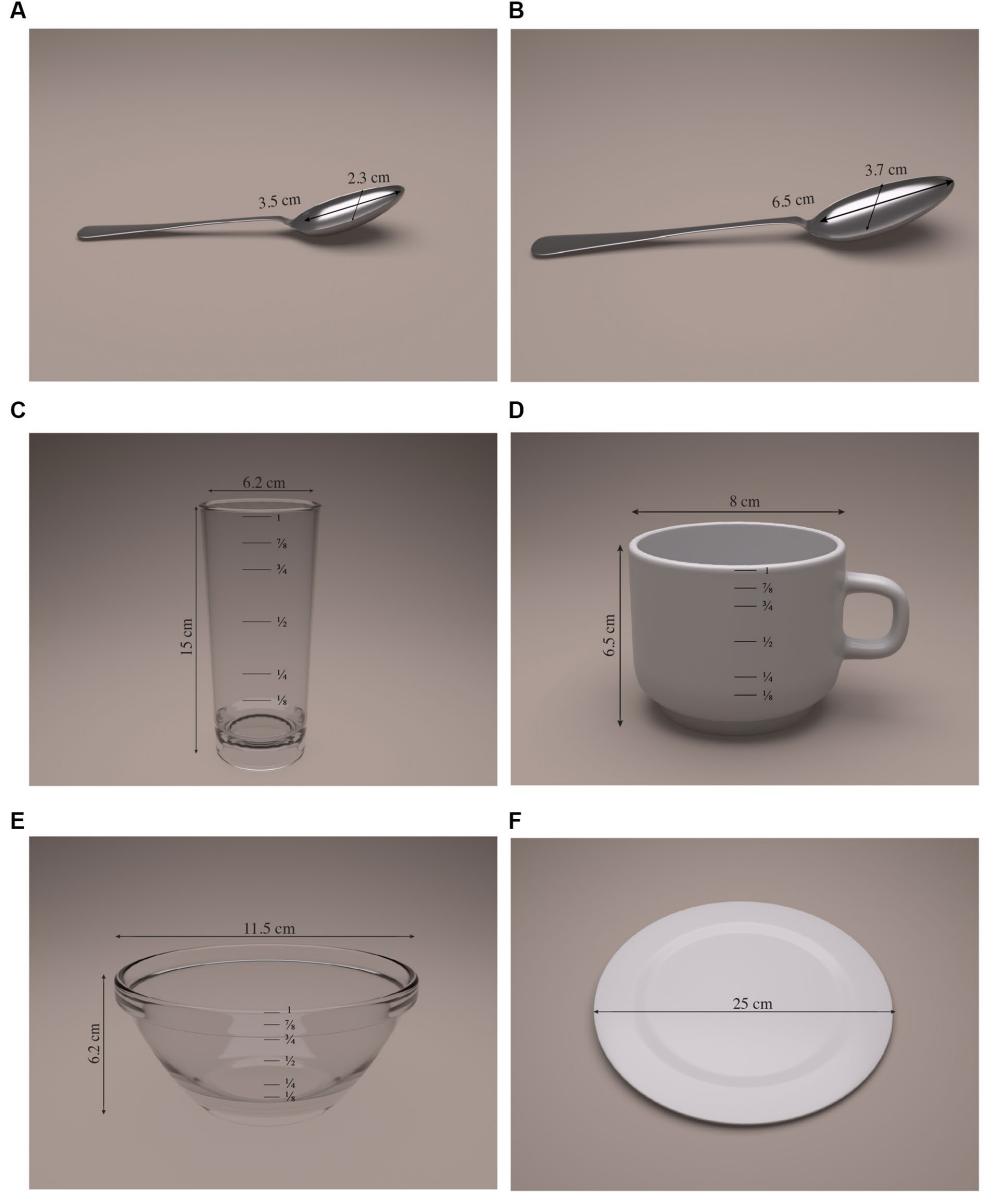
Selected household measurements for the Ecuadorian food exchange list. **(A)** Teaspoon (tsp), 5 mL. **(B)** Tablespoon (Tbsp), 10 mL. **(C)** Large glass, 330 mL. **(D)** Cup, 250 mL. **(E)** Bowl, 320 mL. **(F)** Large plate, 25 cm.

The process of selecting the household measurements most frequently used by Ecuadorian homes made it possible to define the most commonly used sizes. Figure 1 shows the selected household measurements. Additionally, measurements such as length, width, and height were established for those food items that required them.

### 3.2. Definition of the food groups and amounts of the exchange portions

The list of food groups was established according to the predominant macronutrient (carbohydrates, proteins, or total fats). For example, in the group of legumes, proteins were considered the most representative nutrient, while carbohydrates were the most representative for the group of cereals, tubers, and plantains. Finally, 8 general food groups were derived, including each food item’s net weight that met statistical requirements and the equivalent in household measurement. They were organized as follows: cereals, tubers, and plantains; fruit; vegetables; dairy; meat, fish, and eggs; legumes; fats and nuts; and sugars and sugary foods.

Moreover, to differentiate the high, medium, or low content of the predominant macronutrient, certain food groups were also organized into subgroups. For example, in the vegetable group they were classified according to their carbohydrate content into (a) low in carbohydrates (CHO = 5 g) and (b) freely consumable (CHO = 3 g). When necessary, a predominant secondary nutrient was considered in order to organize the subgroups, as in the case of the cereals, tubers, and plantains group where the main macronutrient was carbohydrates and the secondary nutrient was fats, resulting in subgroups with (a) medium or (b) low total fat content; this same criterion was established in the dairy group. Tables 1 2 show examples of the organization of the food items that make up each group, accompanied by their names in Spanish and English, net weight (g), and household measurement. Supplementary Tables S1 and S2 from the supplementary material show the food exchange lists for the group of fats and nuts, and sugars and sugary foods, respectively.

**Table 1:** Food exchange list for the legume group.

Name in Spanish	Name in English	NW (g)	Household measurement
Arveja, grano seco, sin sal, cocida	Pea, mature seed, without salt, cooked	100	4½ heaped tbsp., ½ cup
Arveja, grano seco, cruda	Pea, mature seed, raw	35	3 level tbsp., ^1^/_8_ cup
Chocho, con sal, cocido	Lupine, mature seed, with salt, cooked	60	½ cup
Fréjol, blanco, sin sal, cocido	Bean, white, without salt, cooked	90	½ cup
Fréjol, blanco, grano seco, crudo	Bean, white, mature seed, raw	35	3 level tbsp., ^1^/_8_ cup
Fréjol, negro, sin sal, cocido	Bean, black, without salt, cooked	90	½ cup
Fréjol, negro, grano seco, crudo	Bean, black, mature seed, raw	40	3 level tbsp., ^1^/_8_ cup
Fréjol, rojo, sin sal, cocido	Bean, kidney, without salt, cooked	90	3 full tbsp., ½ cup
Fréjol, rojo, grano seco, crudo	Bean, kidney, mature seed, raw	35	2 full tbsp., ^1^/_8_ cup
Garbanzo, sin sal, cocido	Chickpea, without salt, cooked	90	¾ cup
Garbanzo, grano seco, crudo	Chickpea, mature seed, raw	35	¼ cup, 2 full tbsp.
Haba, grano seco, cruda	Broadbean, mature seed, raw	35	3 level tbsp.
Haba, grano seco, tostada	Broadbean, mature seed, toasted	35	3 full tbsp., ¼ cup
Harina de garbanzo	Chickpea, flour	30	3 heaped tbsp.
Harina de haba	Broadbean, flour	30	1½ heaped tbsp.
Lenteja, grano seco, sin sal, cocida	Lentil, mature seed, without salt, cooked	90	3 heaped tbsp., ¾ cup
Lenteja, grano seco, cruda	Lentil, mature seed, raw	35	3 full tbsp., ¼ cup
Soya, bebida	Soy, beverage	200	78 cup, ¾ glass
Soya, brote	Soy, sprout	75	raw: 1½ cup; cooked: 1 cup
Soya, polvo	Soy, milk powder	30	3 heaped tbsp.

NW, Net weight; S, small unit; M, medium unit; L, large unit.

**Table 2:** Food exchange list for the vegetables group.

Name in Spanish	Name in English	NW (g)	Household measurement
Low in carbohydrates			
Achogcha, cruda o cocida	Balsam-pear (bitter gourd), raw or cooked	100	S: 5 units; M: 2½ units; G:1½ unit
Alcachofa, sin sal, cocida	Artichokes, without salt, cooked	45	½ unit
Alcachofa, leaves y corazón, cruda	Artichokes, leaves, and heart, raw	50	½ unit
Arveja, tierna, enlatada	Pea, canned	35	^1^/_8_ cup
Arveja, tierna, sin sal, cocida	Pea, without salt, raw or cooked	35	¼ cup
Berenjena, sin sal, cocida	Eggplant, without salt, cooked	60	5 slices
Berenjena, cruda	Eggplant, raw	70	7 slices, ½ unit
Brócoli, sin sal, cocido	Broccoli, without salt, cooked	70	S: 20 units; M: 3 units; L: 2 units
Brócoli, crudo	Broccoli, raw	70	S: 27 units; M: 4 units; L: 2 units
Cebolla roja o paiteña, cruda	Onions, raw	85	½ unit
Choclo, dulce, enlatado	Sweet corn, canned	30	2 full tbsp.; ¼ cup
Choclo, amarillo o blanco, en grano, crudo o cocido	Tender corn, yellow or white, raw or cooked	20	2 full tbsp.; ¼ cup
Col blanca, sin sal, cocida	Cabbage, without salt, cooked	95	2 leaves; 1 heaped cup
Col blanca, cruda	Cabbage, raw	100	2 cups
Coliflor, sin sal, cruda o cocida	Cauliflower, without salt, raw or cooked	108	1 cup
Espárrago	Asparagus	125	Raw: 9–10 units; Cooked: 9 units
Espinaca, sin sal, cocida	Spinach, without salt, cooked	120	1 ½ cup
Espinaca, cruda	Spinach, raw	120	3 cups
Frejol, tierno, toda variedad	Tender bean, all varieties	15	Raw: 1 full tbsp.; ^1^/_8_ cup; Cooked: 2 full tbsp.
Haba, tierna, cruda o cocida	Broad beans, immature seeds, raw or cooked	40	S: 28 units; M: 22 units; L: 12 units; ¼ cup
Nabo raíz, crudo o cocido	Turnips, without salt, raw or cooked	90	1½ cup
Palmito, enlatado	Heart of palm, canned	100	¾ cup
Pimiento, rojo	Pepper, red	75	½ unit
Remolacha, sin sal, cocida	Beetroot, without salt, cooked	50	13 cup
Remolacha, cruda	Beetroot, raw	50	½ cup
Sambo, crudo o cocido	Gourd, white, flowered raw or cooked	95	¾ cup
Tomate cherry, tomatillo	Cherry tomato	70	½ cup, 7–8 units
Tomate riñon, rojo	Tomato, red	100	½ cup, 3 slices
Vainita, sin sal, cocida	Bean, without salt, cooked	60	Raw: ¾ cup; Cooked: ½ cup
Zanahoria, jugo	Carrot, juice	50	^1^/_8_ cup, ^1^/_8_ glass
Zanahoria, sin cascara, cruda o cocida	Carrot, peeled, raw or cooked	50	13 cup
Zapallo o calabaza, amarilla	Pumpkin	60	Raw: ½ cup; Cooked: ¼ cup
Zuquinni, crudo o cocido	Squash/courgette, raw or cooked	150	1 full cup
Free consumption			
Acelga, sin sal, cocida	Chard, Swiss, without salt, cooked	80	1 level cup
Acelga, cruda	Chard, Swiss, raw	90	4 heaped cups
Alfalfa, brotes	Alfalfa, sprouts	80	2 ½ cups
Apio, tallos en palitos o cubos	Celery, sticks or cubes, raw	130	Sticks: 1 cup; Cubes: ½ cup
Berros, frescos	Watercress, raw	100	4 heaped cups
Champiñones, sin sal, crudo o cocido	Champignon mushrooms, without salt, raw or cooked	75	5 units
Lechuga, romana, crespa	Lettuce, cos, romaine, raw	100	1 small unit
Lechuga, criolla	Lettuce, green leaf, raw	125	6 leaves
Pepinillo	Cucumber	150	¾ unit; 20 slices; 1 ½ cups
Rábano	Radishes, raw	200	2 full cups
Rúcula	Arugula, raw	85	2 ½ cups

NW, Net weight; S, small unit; M, medium unit; L, large unit.

### 3.3. Average values of macronutrients and energy in the food groups

The average nutritional content of the exchange portions for each food group is shown in Table 3. In addition, the respective subgroups are shown, according to the high, medium, or low content of any of the predominant macronutrients and secondary nutrients, when applicable.

**Table 3:** Nutritional content of exchange portions for each food group, respecting statistical limits.

Food group	*N*	Energy (kcal)		SD	CV (%)	Carbohydrates (g)		SD	CV (%)	Proteins (g)		SD	CV (%)	Total fats (g)		SD	CV (%)
**Cereals, tubers, and plaintains**																	
High in carbohydrates and low in fat	55	150	±	20	14	30	±	4	12	4	±	2	53	1	±	1	95
Medium carbohydrate and medium fat	18	145	±	11	8	20	±	2	12	2	±	1	37	6	±	2	28
Medium carbohydrate and low fat	15	105	±	16	14	20	±	2	9	3	±	1	47	1	±	1	83
**Fruit**																	
Medium carbohydrate	34	90	±	13	15	20	±	3	14	1	±	1	52	0	±	0	119
Low carbohydrate	22	50	±	9	19	10	±	2	19	1	±	1	99	0	±	0	98
Dried fruit	10	95	±	13	14	20	±	4	17	1	±	1	79	0	±	0	125
**Vegetables**																	
Low in carbohydrates	34	30	±	4	16	5	±	0	8	1	±	1	63	0	±	0	73
Free consumption	11	25	±	4	16	3	±	0	10	2	±	1	44	0	±	0	49
**Dairy products**																	
Whole milk, high in fat	3	150	±	2	2	11	±	0	2	8	±	0	3	8	±	0	1
Semi-skimmed, medium fat	2	110	±	7	6	11	±	0	3	8	±	0	0	4	±	1	15
Skimmed, low fat	2	90	±	9	10	12	±	0	2	8	±	0	3	1	±	1	79
Skimmed and high in carbohydrates	2	150	±	7	5	25	±	1	4	6	±	0	4	3	±	1	40
Cheese	18	95	±	21	23	1	±	1	104	7	±	1	10	7	±	3	42
**Meat, fish, and eggs**																	
High in fat	8	180	±	25	14	1	±	2	208	11	±	2	18	15	±	3	19
Medium fat	20	110	±	16	14	0	±	0	242	11	±	2	17	7	±	1	19
Low in fat	55	65	±	11	16	0	±	1	204	11	±	1	10	2	±	1	54
Cold meats	9	100	±	34	35	1	±	1	59	7	±	1	20	7	±	4	52
**Legumes**	20	120	±	15	12	20	±	5	29	9	±	1	9	1	±	2	113
**Fats and nuts**																	
High in fat	19	120	±	18	15	1	±	2	161	1	±	2	168	12	±	2	19
Medium fat	8	75	±	8	11	2	±	2	110	1	±	1	107	7	±	1	16
High in fat and low in carbohydrates	8	140	±	15	11	8	±	4	51	4	±	2	42	10	±	2	20
Medium fat and low in carbohydrates	5	125	±	23	19	12	±	4	28	2	±	1	49	7	±	1	14
**Sugars and sugary foods**	26	40	±	10	23	10	±	2	16	0	±	0	156	0	±	0	196

### 3.4. Photographic record

**Figure 2:**
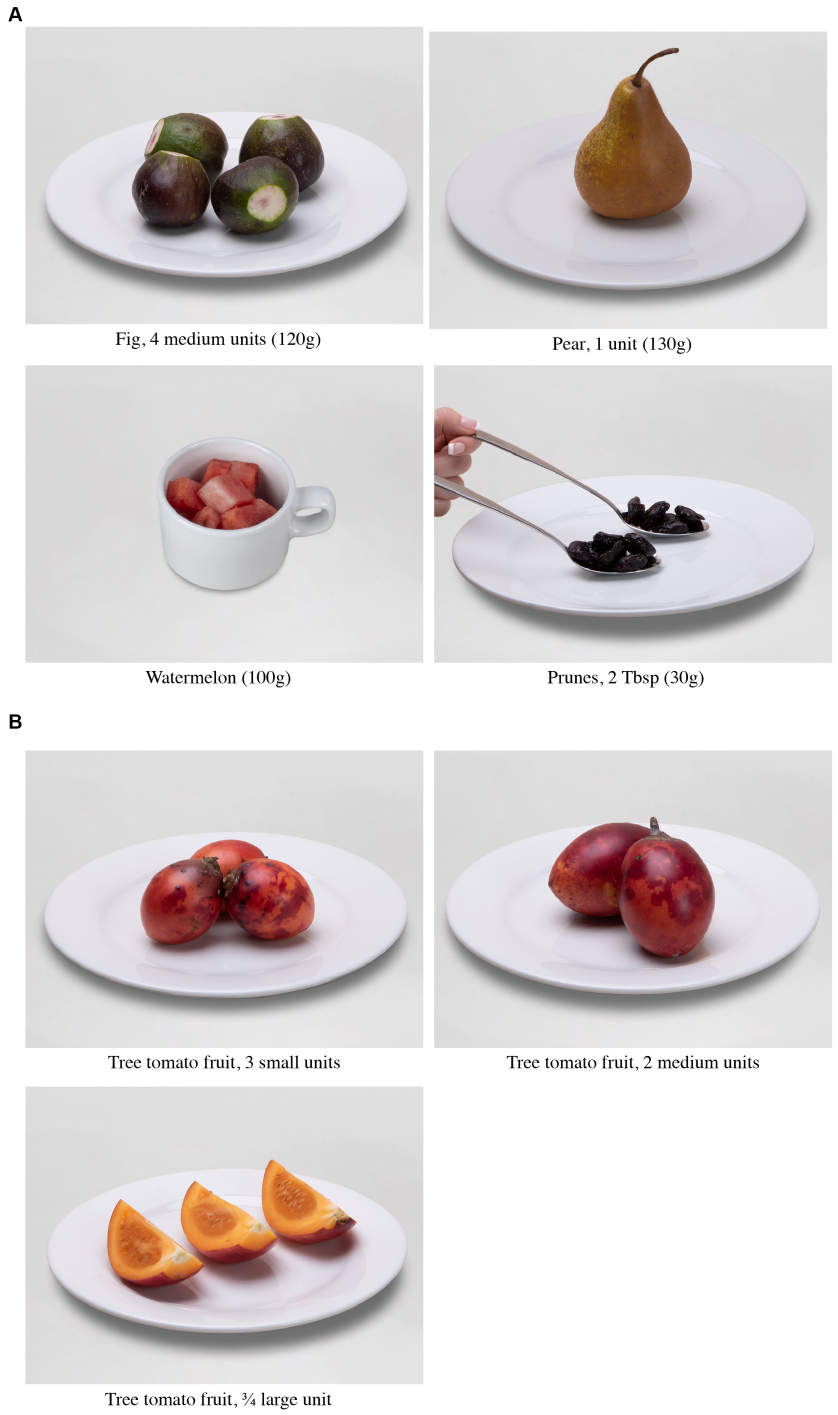
Fruit exchanges according to variety and quantity. **(A)** Variety of fruits. **(B)** Amount based on the size of the food item (small, medium, or large).

A total of 2,600 photographs were recorded since several photographic proofs were needed for each food item, given that between one food item and another the parameters of lighting or framing were adjusted when necessary. The selection and editing process focused on standardizing and/or correcting minor aspects without altering the proportions, colors, or shapes of the images. Finally, 586 photographs were obtained, corresponding to 404 food items, which constitute the photographic record of the food exchange list. Figure 2 shows an example of the exchange portions for the fruit group, considering the variety and quantity of the food items.

## 4. Discussion

Dietary intervention is an essential part of the prevention and treatment of metabolic pathologies, such as chronic non-communicable diseases like diabetes, obesity, or metabolic syndrome, which are becoming more and more widespread, reaching epidemic levels in various countries (32, 33). This situation requires an adequate management of the food portions from each food group that make up a healthy eating pattern. Thus, a food exchange list providing food options that are achievable and sustainable within the culture, preferences, and capabilities of each country’s population enables dietary planning because it includes a list of foods with similar contributions of energy and macronutrients. In this way, the list ensures that the intervention covers the individual or collective nutritional requirements in order to help people adopt appropriate lifestyle modifications related to diet.

To the best of our knowledge, this is the first study to develop a national list of food exchanges for the Ecuadorian population, which includes a diversification in food groups and subgroups, created to improve nutritionists’ effectiveness in planning convenient and culturally sensitive diets and in turn patients’ adherence to their dietary treatment. From now on, a list of food exchanges with 404 foods commonly consumed in Ecuador will be available to be used by nutritionists, dietitians, educators, health professionals, and researchers. The food exchange system has been designed to help convert evidence-based nutrition recommendations into food choices according to Ecuador’s dietary patterns. For the development of the food exchange list, it was first necessary to establish the most commonly used household measurements in the population since these tools allow the patient to manage their diet in an autonomous way. For this reason, the process of defining said measurements was based on the identification of the measurements that are most commonly sold in large-scale vending centers on the one hand, and on the other hand, based on the recommendations of the team of experts in food intake in the different regions of the country. We identified six household measurements that include teaspoon, tablespoon, cup, glass, bowl, and plate (16). Experts such as Marques-Lopes (8, 34) and Jayawardena (12) also recommend defining household measurements based on regular units and, if necessary, distinguishing small, medium, and large units, and accompanying the use of the country’s own household measurements. Following these recommendations, commercial units were included in the Ecuadorian list for packaged foods and for those that require a presentation based on length, width, and height, as in meat and fish. The criterion that allows one to differentiate the size of the food into small, medium, and large, as in the case of vegetables or fruits, was also included. This is an essential step prior to the definition of food quantities.

Since the 1950s to the present day, different lists and food groups have been created, which have been updated as data on the chemical composition of food items have increased and dietary recommendations for the population have been updated. The Ecuadorian list considers eight food groups organized according to the predominant macronutrient (see Table 3) and when necessary, subgroups were created depending on the variability mainly in the fat content, considered as a secondary nutrient in most groups. The introduction of the different subgroups of this list respects statistical inclusion criteria. Given that it is observed that an individual chooses foods for many reasons and the food items selected over the years can make a significant difference in the health of that individual, the diversity of foods included in this list will allow the patient to have different options and greater flexibility in their diet. In addition, the management of local household measurements was added, which is expected to increase the probability of better adherence to specific diets such as those to be applied in patients with non-communicable diseases (3). This type of classification has been established in lists created in Spain (8), Mexico (17), Peru (13), and the United States (18). It should be noted that each country has included subgroups of food items depending on their cultural and dietary context. For instance, Mexico includes a group called “energy-free foods” to refer to sweet and savory spices, e.g., Tabasco sauce; this is because they are consumed frequently and in representative quantities. Spain, on the other hand, includes the subgroup “confectionary and others” to refer to sweet pastry products high in sugar, such as the traditional consumption of croissants. In the more Latin American context, Peru highlights the consumption of the subgroup “stewed beans” to refer to local dishes prepared with dried legumes. In Ecuador, due to the frequent consumption of cereals, tubers, and plantains such as rice, sweet potato, and green plantain, the subgroup “high in carbohydrates and low in fat” was created. On the other hand, groups such as vegetables, dairy products, and meat, fish, and eggs are framed within the criteria of their high, médium, or low content with respect to their primary macronutrients.

The first group presented in this list is that of cereals, tubers, and plantains since these form the basis of the Ecuadorian diet (35). Due to the great diversity of foods rich in carbohydrates, this group was organized into three subgroups according to the CHO content (20–30 g), where their total fat content (1–6 g) was considered as a secondary nutrient since their nature or degree of processing – as happens with bakery products – increases their average total fat content to 6 g ± 2 per exchange portion. This classification was established by taking into account that carbohydrates are consumed as the main source of energy in the population (27); in this way, a list of 88 foods that will allow patients to diversify their diet was created. The needs of the clinical approach where the exchange lists are used were also considered since, depending on the high, medium, or low CHO content, their recording is facilitated, for example in patients with Type 1 or Type 2 diabetes, and gestational diabetes, who need to combine their pharmacological treatment with a contribution and subdivision of CHO in their diet (2, 36). Other countries in the region have also developed their lists by considering the group of cereals, tubers, roots, stewed beans, and plantains as basic food items for the population, while always respecting each nation’s food culture (13–15). With respect to the lists from other countries in the region, we emphasized in our list the inclusion of subgroups that enable people to eat a varied diet while also considering the high, medium, or low content of macronutrients.

The second group of foods where the classification by subgroups was also applied is the dairy group, which was organized by considering its protein content as the primary nutrient, and total fat content as its secondary nutrient, resulting in high-, medium-, and low-fat subgroups (Total fat: 1–8 g). Given the existence of low-fat products that are rich in CHO, we saw the need to include an appropriate subgroup since the average CHO content of these products reaches 25 g ± 1, resulting in a caloric intake equal to a high-fat dairy product. These low-fat, CHO-rich products are generally associated with a higher content of simple sugars, and their inclusion in dietary plans can influence the nutritional education of patients who require strict CHO control, such as patients with diabetes. Moreover, in this group it was necessary to include a subgroup exclusively for the types of cheese sold in the Ecuadorian market (n:18), ordering the list from highest to lowest according to their fat content, with an average fat intake of 7 g ± 3. The exchange portion sizes were measured in terms of length, width, and height for those types of cheese that are not sold in regular sizes. This kind of classification associated with dairy products has been applied around the world (8, 13, 15, 34) since the consumption of these products is increasing; therefore, it is necessary to differentiate those with higher fat and sugar content, as established by Marques-Lopes et al. (8) in Spain, where they also include subgroups related to the consumption of sweetened dairy desserts, which is very common in that country (8). For the Ecuadorian list, this type of food was not considered since there is not a significant demand for them and they are not yet cataloged as frequently consumed foods.

Given the diversity of protein-rich foods, such as meat, fish, and eggs, commonly consumed by the Ecuadorian population, it was necessary to organize them into four subgroups also according to a secondary macronutrient, in this case total fat content (Total fat: 2–15 g). This is because evidence has indicated that fat intake should not exceed 35% of the requirement, since fats can play a positive or negative role in the prevention or treatment of diseases (37, 38). Along these lines, a single group was also determined for foods with medium and high fat contents, defined as fats and nuts (Total fat: 7–12 g), which includes food items like oils, solid fats (e.g., butter), nuts, and seeds. Thus, the list will allow the patient to make more suitable choices.

Finally, the groups of fruits, vegetables, legumes, and sugars and sugary foods were organized only according to their primary macronutrient, and the carbohydrate content was established for all of them since no statistical variability was found that would show the need to create subgroups of food items according to a secondary nutrient. For each item, the most appropriate household measurement was used according to the form of consumption.

Thus, within a food group, one exchange is approximately equal to another in terms of energy, carbohydrates, protein, and total fat, and can be exchanged for any other food in the same list. To achieve this goal, the estimation of the coefficient of variation was applied, which had to be CV < 30%, evidencing that it was strictly complied with for the primary macronutrients in all groups. The cold meats subgroup is the only one that exceeds this limit (CV = 35%) in caloric value; however, thanks to the Z parameter, this caloric variability does not seem to be representative, since all the foods in this subgroup show a Z value equivalent to ±2. This variability may be due to the fact that in these food items, the regular and commercialized size was respected, instead of a size based on the use of household measurements, and added to this, the effect of the nutritional compositions of the products themselves was taken into account.

Therefore, the Ecuadorian list can be used in clinical practice, for example in the case of patients with diabetes since local food items were included in the general groups, such as fruit, legumes, and cereals, tubers, and plantains, and these were organized on the basis of 10–30 g of CHO per exchange portion, which facilitates carbohydrate counting. This organization allows the nutritionist to evenly distribute the CHO count in the patient’s daily menu. Protein quantification is also essential in clinical practice, especially in patients with differentiated requirements, such as renal, burn, or malnourished patients (3), as well as in special situations in healthy populations with high protein-energy requirements, such as athletes (39). In this regard, the Ecuadorian list also organizes the food groups according to their average protein contribution (6–11 g of protein per exchange portion). In particular, the information for meat, fish, and seafood, dairy products, and legumes will allow both patients and athletes to define their food plans with a greater diversity of options.

For each exchange portion, a photographic record was taken according to the most common forms of consumption, that is, the same exchange can be presented in a cup, glass, or units consumed depending on the size– small, medium, or large. This will allow a nutritionist to add a visual tool to nutrition education because in several studies it has been shown that the use of photographs improves the perception of the size of the portion consumed (40, 41).

This food exchange list was created based on the food context of the Ecuadorian population. Yet this study has certain limitations, as outlined below. One of the limitations is related to the sources where the information on the nutritional composition of foods was obtained. Most of these data were taken from composition tables from other countries because in Ecuador the last reference of a National FCT where bromatological analyses have been performed dates back to 1965 and to correctly estimate intake, it is necessary to have an updated Ecuadorian FCT. The Ecuadorian table (1965) analyzed very few food items and presented non-updated analytical methods, so the latest updates of the FCT in Ecuador have been carried out by applying using data borrowed from other FCTs or databases in the region. It is known that composition has different factors that can modify it, that it varies from one geographical area to another, according to the analytical method applied or even depends on the cooking techniques used for the food (42). Nonetheless, it has been possible to observe less variability in the macronutrients of natural or minimally processed foods. Therefore, to compile the Ecuadorian list, we searched for these food groups in the most complete sources in the Latin American context, which encompass most of the foods consumed in Ecuador and often furnish its food database. That is why the Central American FCT developed by INCAP has been taken as a regional reference. In addition, the nutritional information was complemented with small national databases, such as the one produced in Cuenca, Ecuador, or for more processed or ultra-processed food items the nutritional information was obtained directly from the food labeling provided by the food industry. The USDA database allowed us to complete the nutritional composition for more processed food items, i.e., fast food. Therefore, the list should be used with caution if the objective in planning is to consider the content of micronutrients since many of the foods might be fortified or supplemented.

Another limitation is not including traditional cuisine in the exchange list, such as soups (e.g., potato locro) or rice (e.g., rice with seafood). The reason for this is the absence of official records of nutritional composition analysis measured in laboratories that inform one of their standardized contributions, hence they simply could not be included; this could limit their inclusion in meal plans or food guides. Nonetheless, an extensive list of foods or ingredients with which food alternatives can be generated according to the region is included. For example, exchanges for cooked rice, cooked shrimp, or cooked fish are presented, which facilitates the combination and therefore the generation of new recipes. Another limitation that the list may present is non-standardization, especially considering critical micronutrients in the Ecuadorian population (e.g., iron, zinc, calcium, and vitamins A and C). Such an analysis should be performed in future studies since their relationship with typical health problems in the country has been demonstrated. Despite these limitations, this study provides health professionals and consumers with a system of food exchanges that facilitates menu planning, as it can be used in individualized dietary planning or collective nutritional.

This food exchange list targeted at the Ecuadorian population is the first list to be made using a process that respects statistical criteria, thus categorized as a good source of information on macronutrient and energy content divided into eight major food groups, all supported by their photographic record. It provides detailed information on food quantities using household measurements to allow dietitians, nutritionists, researchers, and other health professionals to develop culturally sensitive meal plans with healthier choices tailored to clinical or population needs. Diets planned from exchange lists that include local food items are more likely to be successfully implemented, helping to reduce problems related to adherence, limited food choices, or exchange portion sizes that are unrealistic for the country context.

## Data availability statement

The raw data supporting the conclusions of this article will be made available by the authors, without undue reservation.

## Author contributions

AMC-T, MH-F, and GV-R designed the project, were involved in data and sample collection, and wrote the manuscript. AMC-T, and MH-F participated in the analyses and interpretation of data after performing the statistical analyses. All authors performed a critical review of the final manuscript, contributed to the article, and approved the submitted version.

## Funding

AMC-T, and MH-F extend their thanks to the Escuela de Salud Pública, Universidad San Francisco de Quito (USFQ) for their grant. GV-R would like to thank the Escuela de Comunicaciones y Artes Contemporáneas, Universidad San Francisco de Quito, for their grant. The funders had no role in the study design, data collection, data analysis, decision to publish, or preparation of the manuscript.

## Conflict of interest

The authors declare that the research was conducted in the absence of any commercial or financial relationships that could be construed as a potential conflict of interest.

## Publisher’s note

All claims expressed in this article are solely those of the authors and do not necessarily represent those of their affiliated organizations, or those of the publisher, the editors and the reviewers. Any product that may be evaluated in this article, or claim that may be made by its manufacturer, is not guaranteed or endorsed by the publisher.

## Supplementary material

The Supplementary material for this article can be found online at: https://www.frontiersin.org/articles/10.3389/fnut.2023.1219947/full#supplementary-material
