# Supplementary material for: Food exchange list based on macronutrients: adapted for the Ecuadorian population
Source: Front Nutr. 2023 Aug 10;10:1219947. doi: 10.3389/fnut.2023.1219947 (PMC10450507; doi:10.3389/fnut.2023.1219947)
Supplement: Supplementary file 1 [file Data_Sheet_1.docx]

**Table 1S: Food exchange list for fats and nuts group.**

| **Name in Spanish** | **Name in English** | **NW**  **(g)** | **Household Measurement** |
| --- | --- | --- | --- |
| **High in fats** |  |  |  |
| Aceite de aguacate | Avocado Oil | 15 | 2 full tbsp. |
| Aceite de ajonjolí | Sesame Oil | 15 | 2 full tbsp. |
| Aceite de almendra | Almond Oil | 10 | 1 full tbsp. |
| Aceite de canola | Canola Oil | 15 | 2 full tbsp. |
| Aceite de coco | Coconut Oil | 15 | 2 full tbsp. |
| Aceite de girasol | Sunflower Oil | 15 | 2 full tbsp. |
| Aceite de maíz | Corn, Oil | 15 | 2 full tbsp. |
| Aceite de nuez | Walnut, Oil | 10 | 1 full tbsp. |
| Aceite de oliva | Olive, Oil | 15 | 2 full tbsp. |
| Aceite de palma | Palm, Oil | 15 | 2 full tbsp. |
| Aceite de soya | Soyabean, Oil | 15 | 2 full tbsp. |
| Chicharrón de cerdo | Pork, rind | 20 | 2 full tbsp. |
| Coco maduro, pulpa | Coconut, ripped, pulp | 30 | ^1^/_8_ cup |
| Crema de leche, espesa | Whipped cream | 30 | 2 full tbsp. |
| Maní o cacahuate, con sal, tostado | Peanut, salted, toasted | 20 | 2 full tbsp. |
| Maní o cacahuate, con sal, frito | Peanut, salted, oil-roasted | 20 | 2 full tbsp, ¼ cup |
| Manteca de cerdo | Lard | 10 | 1 full tbsp. |
| Nuez de nogal | Walnut | 15 | Whole: 3 units, Peeled: 6 halfs, 2 full tbsp. |
| Queso crema | Cheese, cream | 30 | 1 heaped tbsp. |
| **Medium fat** |  |  |  |
| Aceituna verde, sin hueso, envasada | Green olive, ripe, canned | 60 | ½ cup, 20 units |
| Aguacate, serrano | Avocado, native | 50 | 1 unit |
| Aguacate, común | Avocado, common | 50 | ¼ unit |
| Chontacuro o mayón, frito | Chontacuro, fried | 25 | 1 unit |
| Crema agria | Sour cream | 50 | 2 full tbsp. |
| Mantequilla, con sal o sin sal | Butter, with or without salt | 10 | 2 full tsp. |
| Margarina, 20% grasa, con sal | Margarine, 20% fat, salted | 40 | 2 heaped tbsp. |
| Margarina, 80% grasa, con sal | Margarine, 80% fat, salted | 10 | 1 level tbsp. |
| **High in fats and low in carbohydrates** |  |  |  |
| Almendra | Almond | 25 | 2 full tbsp., ¼ cup, 30 units |
| Mayonesa, comercial, con sal | Mayonnaise, salted | 30 | 2 full tbsp. |
| Semilla de ajonjolí | Sesame seed | 20 | 2 heaped tbsp., ^1^/_8_ cup |
| Semilla de chía | Chia seed | 30 | 3 full tbsp., ¼ cup |
| Semilla de girasol | Sunflower seed | 20 | With shells: ½ cup, Without shells: 2 full tbsp. |
| Semilla de linaza | Flax seed | 25 | 2 full tbsp., ¼ cup |
| Semilla de pistacho, con sal tostada | Pistachio nut, salted, toasted | 25 | With shells: 3 full tbsp., ½ cup  Without shells: 2 full tbsp., ¼ cup |
| Semilla de sambo, sin sal tostada | Pumpkin seed, salted, toasted | 30 | 3 full tbsp., $\frac{1}{3}$ cup |
| **Medium fat and low in carbohydrates** |  |  |  |
| Chocolate con leche, *Crunch de Nestle* | Chocolate, with milk, *Crunch by Nestle* | 30 | ½ unit |
| Cocoa, polvo | Cocoa, powdered | 15 | 2 full tbsp. |
| Crema de cacao y avellana, *Supermaxi* | Cocoa and hazelnut cream, *Supermaxi* | 20 | 1 full tbsp. |
| Helado de crema | Ice cream | 60 | 1 unit |
| Hojuelas de coco, pulpa seca, azucaradas | Coconut meat, dehydrated, sweetened | 20 | 2 heaped tbsp., $\frac{1}{3}$ cup |

NW: Net weight

**Table 2S: Food exchange list for sugars and sugary foods group.**

| **Name in Spanish** | **Name in English** | **NW**  **(g)** | **Household Measurement** |
| --- | --- | --- | --- |
| Azúcar, blanca | White sugar | 10 | 1 full tbsp. |
| Azúcar, morena, | Brown sugar | 10 | 1 full tbsp. |
| Caña de azúcar | Sugarcane | 10 | 2 pieces |
| Caña de azúcar, jugo | Sugarcane, juice | 50 | ^1^/_8_ glass |
| Caramelos o confites, duros o suaves | Candy, hard or soft | 10 | 3 units |
| Cerezas, en conserva | Cherires, syrup, canned | 50 | 10 units |
| *Chocolisto*, polvo | *Chocolisto,* powdered | 10 | 1 heaped tbsp. |
| Dulce de guayaba | Guava sweet | 10 | ½ unit |
| Dulce de leche | Dulce de leche | 10 | 1 level tbsp. |
| Espumilla | Espumilla | 15 | 1 heaped tbsp. |
| Flan de vainilla | Custard, vanilla flavor | 50 | 1 piece |
| Gaseosa, cola negra, con cafeína | Carbonated drink, soda, with caffeine | 100 | $\frac{3}{8}$ cup, ¼ glass |
| Gelatina, todo sabor, con azúcar | Jelly, all flavors, with sugar | 70 | ¼ cup |
| Chicle, con azúcar | Chewing gum, with sugar | 10 | 5 units |
| Helado de agua, sabor artificial | Water ice cream, artificial flavor | 50 | 1 unit |
| Helado de paila, distintas frutas, artesanal | De paila ice cream, with fruits, artisan | 40 | 1 unit |
| Higo en almíbar, envasado | Fig, in syrup, canned | 30 | 1 unit |
| Jugo procesado, sabor a naranja, *del Valle* | Processed juice, orange flavor*, del Valle* | 100 | $\frac{3}{8}$ cup, ¼ glass |
| Leche condensada, enlatada | Condensed milk, canned | 20 | 3 full tbsp. |
| Malta, *Pony Malta* | Malt drink, *Pony Malta* | 100 | $\frac{3}{8}$ cup, ¼ glass |
| Mermelada, todos los sabores | Jam, all flavors | 15 | 1 level tbsp. |
| Miel de abeja | Honey | 15 | 1 level tbsp. |
| Miel de maple | Maple syrup | 15 | 1 full tbsp. |
| *Milo*, polvo | *Milo*, powdered | 15 | 1 heaped tbsp. |
| Panela o raspadura | Panela | 10 | 1 heaped tbsp. |
| Salsa de tomate o ketchup | Kétchup | 30 | 2 full tbsp. |

NW: Net weight
